# Thyrotomy

**Published:** 1902-04-26

**Authors:** 


### THYROTOMY.

At a recent meeting of the Medical Society a paper was read by Mr. Marmaduke Sheild on a series of external operations on the larynx. Among the conditions which he had thus dealt with were obstructions of the glottis by cicatricial tissue after fracture of the larynx, syphilitic stenosis, papillomatous growths, impacted foreign body (bone), and laryngeal cancer. In the performance of the operations he strongly advises that, instead of making separate incisions for the thyrotomy and the preliminary tracheotomy, both larynx and trachea should be exposed by one longitudinal incision from above the thyroid cartilage to a point $1\frac{1}{2}$ inch below the cricoid. The hæmorrhage is always profuse, but in this large wound the veins are easily and quickly caught with pressure forceps—an agreeable contrast with the perpetual "welling up" of venous blood which takes place with smaller incisions. The second stage of the operation is that of opening the trachea, which is done deliberately below the cricoid cartilage, and the tube is then inserted. Mr. Sheild has the tube covered with compressed sponge, which is puffed with iodoform, and after its insertion he allows eight minutes to elapse to allow the sponge to swell and the anæsthetist to adapt himself to the new conditions of administration. The thyroid may be divided by a very fine saw or by strong scissors curved on the flat. If possible, the extreme upper border of the cartilage should be left intact so as not to interfere with the petiolus of the epiglottis. On the cut margins being thoroughly everted and retracted by an assistant the growth or foreign body at once comes into view in a remarkably clear manner, and it is to be noted that, if a growth, it is always more extensive than had appeared from laryngeal examination. At this stage one has to decide as to the extent of the operation which must be undertaken. The operation is usually done for what may be termed potential epithelioma, warty growth with infiltration at the base in adults, or for actual epithelioma of one or other of the vocal cords, and the tissues from which the growth springs have generally to be pretty freely removed. By painting the growth with a 20 per cent. solution of cocaine, to which is added 10 grains of freshly prepared suprarenal extract dissolved in half a drachm of cold water, the troublesome local oozing is strikingly diminished. A small and carefully purified Turkey sponge, with a piece of silk attached, is to be pressed into the trachea at the lower angle of the laryngeal wound. In removing the growth everything is to be sacrificed down to the cartilage beneath. Pressure is made on the wound inside the larynx with minute sponges wrung out in spirits of turpentine. Then the surface of the wound is curetted, and finally the galvano-cautery at a cherry red heat is applied so as to render the extirpation as free as possible. At the same time this checks all oozing. The divided thyroid is then united with strips of kangaroo tendon, and the outer incision with fishing gut and horse-hair right down to the tracheotomy tube. As a rule, Mr. Sheilds removes the tube as soon as ever the patient has recovered his "coughing reflex." After that any blood which may enter the air passages is easily expelled by coughing. The tube wound, however, is not closed till about the fourth day, when the edges are carefully drawn together by strapping. After the operation the patient is kept in the sitting position in a steam tent, and occasionally inhales the vapour of creosote. No use of the voice is permitted, and although patients swallow with surprising ease after this operation, rectal alimentation is to be employed for a couple of days, after which meat lozenges may be sucked. The larynx is to be sprayed with "Listerine" and tepid water, equal parts, three times a day. After these operations the ultimate recovery of the vocal functions is often surprising, though the intonation is always hoarse. But the voice must not be used for a full month after the operation, and then only in a quiet whisper.

